# Control of mesenchymal stem cell biology by histone modifications

**DOI:** 10.1186/s13578-020-0378-8

**Published:** 2020-02-03

**Authors:** Jianhan Ren, Delan Huang, Runze Li, Weicai Wang, Chen Zhou

**Affiliations:** Guanghua School of Stomatology, Hospital of Stomatology, and Guangdong Provincial Key Laboratory of Stomatology, Sun Yat-sen University, 56 Lingyuanxi Road, Guangzhou, 510055 China

**Keywords:** Epigenetics, Histone modifications, Mesenchymal stem cells, Cell differentiation, Cellular senescence, Cell biology

## Abstract

Mesenchymal stem cells (MSCs) are considered the most promising seed cells for regenerative medicine because of their considerable therapeutic properties and accessibility. Fine-tuning of cell biological processes, including differentiation and senescence, is essential for achievement of the expected regenerative efficacy. Researchers have recently made great advances in understanding the spatiotemporal gene expression dynamics that occur during osteogenic, adipogenic and chondrogenic differentiation of MSCs and the intrinsic and environmental factors that affect these processes. In this context, histone modifications have been intensively studied in recent years and have already been indicated to play significant and universal roles in MSC fate determination and differentiation. In this review, we summarize recent discoveries regarding the effects of histone modifications on MSC biology. Moreover, we also provide our insights and perspectives for future applications.

## Background

Mesenchymal stem cells are multipotent cells that can differentiate into various lineages whose nomenclature remains inconsistent. Apart from “mesenchymal stem cells”, MSCs are also called “mesenchymal stromal cells”, “bone marrow stromal cells” (BMSCs) and “multipotent stromal cells” [1]. Friedenstein originally discovered MSCs in mouse bone marrow [2]. Since then, MSCs have been isolated from most types of mesenchymal tissue, including skeletal muscle tissue, adipose tissue, umbilical cord tissue, placenta tissue, liver tissue, skin tissue, synovial membranes, dental pulp, periodontal ligaments, cervical tissue, amniotic fluid, lung tissue, and dermal tissue [3].

According to the International Society for Cellular Therapy (ISCT) [4], MSCs must be positive for CD105, CD90, and CD73 while negative for CD45, CD34, CD14 or CD11b, CD79α or CD19, and HLA class II. In addition, MSCs must be able to differentiate into osteocytes, chondrocytes and adipocytes in vitro under differentiating conditions [5]. MSCs can also transdifferentiate into different types of cells from other germ layers, including neurons, epithelial cells, cardiomyocytes, hepatic cells and islet cells [6–10], meaning that they can be used to repair or regenerate various tissues, such as cartilage, bone, and adipose tissue [11]. Moreover, MSCs possess anti-inflammatory and proinflammatory capabilities, enabling them to regulate the immune response [12–14]. MSCs are regarded as immune-privileged because they express MHC-I at low levels and are negative for MHC II and T-cell costimulatory molecules. Compared to embryonic stem cells (ESCs) and induced pluripotent stem cells (iPSCs), MSCs have low tumorigenicity [15]. These advantages make them promising candidates for the treatment of a wide range of diseases, including myocardial infarction, graft-versus-host disease (GvHD), acute respiratory distress syndrome (ARDS), amyotrophic lateral sclerosis, and chronic kidney disease, among others [16–20].

Given the great clinical and scientific significance of MSCs, understanding of their biological characteristics in vivo and in vitro, including their proliferation, differentiation, cellular senescence and responses to abnormal microenvironments, is urgently needed. In this review, we systemically summarize the most recent progress in the field of histone modification-mediated control of MSC biological processes and our perspectives on the latest advancements.

Histone modification refers to posttranslational modification of histones. Most histone modifications involve sites within the first 30 amino acids of the N-terminal domains of histones (also known as histone tails), such as H3K4, H3K9 and H3K27 [21]. There are many different kinds of histone modifications, including acetylation, methylation, phosphorylation, ubiquitylation, SUMOylation, and proline isomerization [22].

Histone methylation, which is mediated by histone methyltransferases (HMTs) and histone demethylases (HDMs), mainly influences lysine and arginine residues on the histone side chains; through this process, lysine can be mono-, di- or trimethylated. Different histone methylations affect gene transcription in different ways in cooperation with other identifying proteins [23–25]. Histone acetyltransferases (HATs) and histone deacetylases (HDACs) are both enzymes that regulate histone acetylation on lysine residues, but they produce opposite results [26, 27]. In addition, proteins that contain specialized binding regions, such as bromodomains, are termed readers [28]; these proteins can recognize and bind to acetylated lysine residues, thus interacting with specific histone modifications [29].

As mentioned above, many types of histone modifications exist apart from methylation and acetylation. The major enzymes and effector molecules corresponding to these modifications are summarized in Table 1.

**Table 1:** Major histone modification writers, erasers and readers

Type	Major member	Modification/identification site	Function
Writers			
Histone acetylation	GCN5	H3K9, H3K14, H3K18, H3K36, H4K8	Transcriptional activation
	p300/CBP	H2AK5 H3K18, H3K27 H4K8, H4K12	Transcriptional activation
	Tip60	H2AK5 H3K14 H4K5, H4K8, H4K12, H4K16	Transcriptional activation
Histone methylation	SUV39h1 SUV39h2	H3K9	Transcriptional silencing
	G9a	H3K9	Transcriptional repression
	SETDB1	H3K9	Transcriptional repression
	EZH2	H3K27	Transcriptional silencing
	SETD2	H3K36	Transcriptional elongation
	CARM1	H3R17, H3R26, H3R42	Transcriptional activation
Histone phosphorylation	Aurora B	H3S10, H3S28	Transcriptional activation
Histone ubiquitylation	Ring1B	H2A	Transcriptional repression
	Rad6	H2B	Transcriptional elongation
Erasers			
Histone methylation	LSD1	H3K4, H3K9	Transcriptional activation/repression
	KDM4B	H3K9	Transcriptional activation
	KDM6B	H3K27	Transcriptional activation
	JMJD1C	H3K9	Transcriptional activation
Histone acetylation	HDAC1	H2A, H2B, H3, H4	Transcriptional repression
	HDAC4	H2A, H2B, H3, H4	Transcriptional repression
	HDAC6	H2A, H2B, H3, H4	Transcriptional repression
	HDAC8	H2A, H2B, H3, H4	Transcriptional repression
Histone phosphorylation	PP1	H3S10, H3S28	Transcriptional repression
Histone ubiquitylation	Ubp8	H2B	Transcriptional activation
Readers			
Acetyl-lysine binding domains	BRD2	Acetylated lysine	Chromatin remodeling
Methyl-lysine binding domains	Brpf1 HP1 L3MBTL1	Methylated lysine	

## Regulatory roles of histone modifications in MSC differentiation

**Fig. 1:**
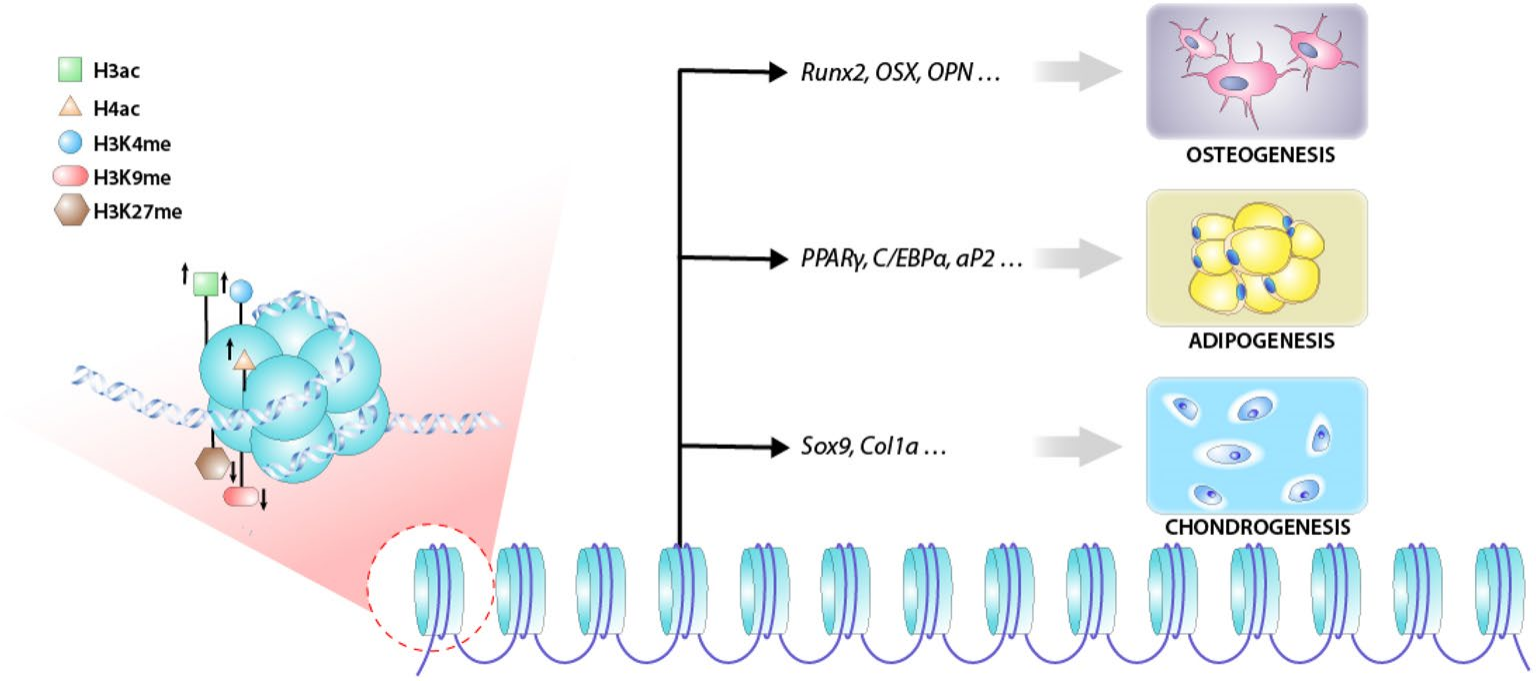
MSC fate determination is associated with histone modifications on specific regions of lineage-specific genes

**Fig. 2:**
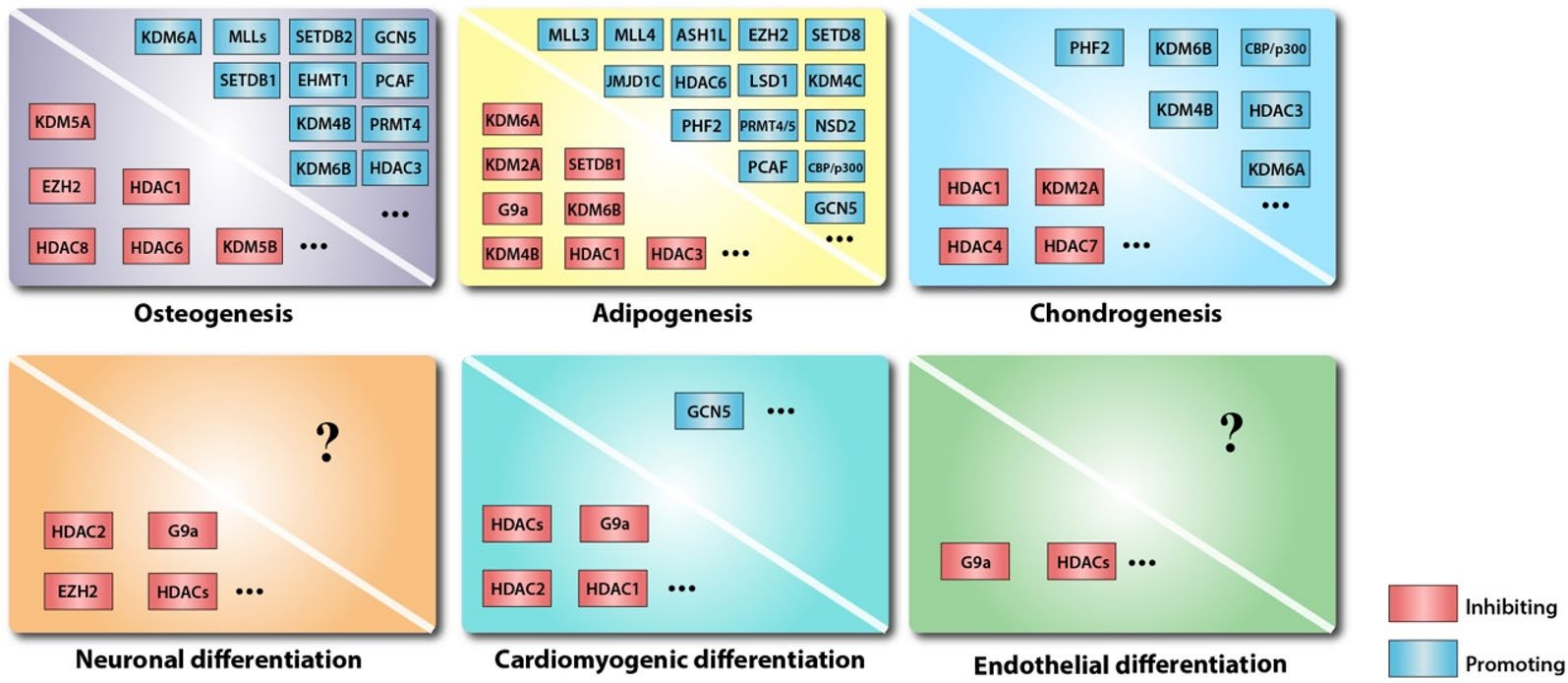
The various roles of histone-modifying enzymes in determining different MSC fates

MSC differentiation is a complex process regulated by various signaling pathways with intricate crosstalk [30–33] in which all types of epigenetic and transcriptional regulation are involved [34]. Early studies focused on how external stimuli and internal transcription factors modulate the differentiation of MSCs; however, recent studies have revealed the roles of histone modifications in this process [35]. For example, it has been well established that histones on the promoter regions of master transcription factors associated with cell fate commitment, such as *RUNX2* and *OSX* in osteogenic differentiation, *PPARG* and *CEBPA* in adipogenic differentiation [36] and *SOX9* in chondrogenic differentiation (Fig. 1), are dynamically modified [37]. Histone modification changes gene expression and thus strongly influences the fate commitment of MSCs. The roles of histone-modifying enzymes are described below and are summarized in Fig. 2.

### Roles of histone modifications in MSC osteogenic, adipogenic and chondrogenic differentiation

Bone formation involves proliferation of MSCs, differentiation of MSCs into progenitor cells and eventually osteoblasts, and secretion of bone matrix by osteoblasts. MSC osteogenic differentiation is critical to bone formation [38], and impairment of the osteoblast differentiation potential of MSCs can lead to various diseases, such as osteoporosis, osteopetrosis, osteopenia, and oculofaciocardiodental (OFCD) syndrome [39–41]. Recent studies profiling the genome-wide patterns of different histone marks have revealed relationships between histone modifications and osteogenic differentiation of MSCs. For example, Meyer and colleagues found that the histone patterns of osteogenic differentiated MSCs are similar to those of undifferentiated MSCs by ChIP-seq and RNA-seq analyses, suggesting an intrinsic preference of MSCs for osteogenic differentiation [36]. Tan and colleagues noted that H3K9ac marks on gene promoter regions globally decrease but that H3K9me2 marks globally increase during osteogenic differentiation through ChIP-on-chip and expression microarray analyses [42].

Adipogenesis is another important process of MSCs. Adipogenic differentiation correlates with the formation of adipose tissue, which is a critical regulator of energy homeostasis, metabolism and immunity [37]. Therefore, disruption of MSC differentiation potential results in abnormal accumulation of adipose tissue [43]. Unlike osteogenic differentiated MSCs, adipogenic differentiated MSCs have a significantly different histone pattern than undifferentiated MSCs [36].

Chondrogenesis is a critical physiological process for cartilage formation. Cartilage formation is essential not only during embryogenesis but also during bone tissue repair or the pathological processes of diseases such as osteoarthritis (OA) [44]. A previous study used genome-wide ChIP and deep sequencing analyses to examine histone mark changes in specific genes during chondrogenic differentiation [45]. The active marks H3K4me3, H3K9ac and H3K36me3 were enriched on upregulated chondrogenic signature genes, including *COL2A1*, *COL9A2*, and *ACAN*, among others, whereas the levels of the repressive mark H3K27me3 were decreased on these genes. In contrast, H3K4me3, H3K9ac and H3K36me3 levels were decreased, while H3K27me3 levels were slightly increased, on the genes associated with MSC traits, such as *CXCL12* [46].

To further clarify the exact roles of histone modifications in these various MSC processes, we will discuss how specific histone marks and histone-modifying enzymes impact the differentiation of MSCs.

#### H3K4 methylation

Methylation of H3K4 is associated with an active or poised transcription state and is found in the promoters of most genes [47–49]. Some previous studies have implied that H3K4me3 levels may be associated with osteogenesis. An initial study reported that depletion of *Hoxa10*, a BMP2-inducible gene, impairs osteogenesis by decreasing H3K4 methylation [50]. Consistent with this report, some HDMs that specifically demethylate H3K4 have been identified to directly influence MSC osteogenic differentiation. In addition, recent studies have proven the negative effects of the HDMs KDM5A and KDM5B on the osteogenic differentiation of MSCs [51, 52]. Knockdown of KDM5A or KDM5B increases H3K4me3 levels in the promoter region of *Runx2* and results in upregulation of osteogenic differentiation. Odontoblasts are cells that secrete dentin during tooth development. These cells differentiate from MSCs under the control of various signaling pathways, including the Wnt, Bmp, Shh and Notch pathways [53–55]. Zhou and colleagues recently found that mixed lineage leukemias (MLLs, H3K4me3 methylases) and KDM6A/B (H3K27me3 demethylases) play essential roles in the expression of *Wnt5a*, a member of the Wnt ligand family, as well as in odontogenic differentiation [56].

In addition to the promoters of genes associated with osteogenesis, those of adipose tissue-specific genes such as *apM1* exhibit increased H3K4me3 levels in MSCs during adipogenic differentiation [57]. The H3K4 methyltransferases MLL3 and MLL4 are components of the ASC-2 complex (ASCOM), which acts as a transcriptional coactivator of PPARγ and C/EBPα. Mice expressing inactivated MLL3 or MLL4 have less fat than mice expressing the activated versions of these proteins, and mouse embryonic fibroblasts isolated from the inactivated MLL3/MLL4-expressing mice exhibit lower adipogenic differentiation potential, suggesting the positive roles of MLL3 and MLL4 in adipogenesis [58]. A recent study also reported that silencing of *Ash1l*, an H3K4 methyltransferase, promotes adipogenesis while suppressing osteogenesis and chondrogenesis by affecting certain master transcription factors. Intriguingly, both ASH1L and H3K4me3 are present at the transcription start sites (TSSs) of *Osx*, *Runx2* and *Sox9*, whereas only H3K4me3 is present at the TSS of *Pparg.* Therefore, silencing of *Ash1l* downregulates H3K4me3 levels on the *Osx* and *Runx2* promoters but not on the *Pparg* promoter, consistent with the impact of *Ash1l* silencing on differentiation [59]. Furthermore, knockdown of KDM2A upregulates SOX2 and NANOG expression by increasing H3K4me3 on the promoters of these genes and thus enhances both adipogenic and chondrogenic differentiation potential in human stem cells from the apical papilla (SCAPs) [60].

#### H3K9 methylation

H3K9 dimethylation and trimethylation are both repressive histone modifications and are mainly catalyzed by G9a/GLP and SUV39H1/2, respectively, mediating the formation of heterochromatic regions through interaction with HP1 [61, 62]. Knockdown of ESET or SETDB1, a H3K9 methyltransferase, causes bone defects in mice [63]. Mechanistically, the impairment of osteogenic differentiation results from histone methylation-induced aberrant expression of *Runx2*. Euchromatin HMT1 (EHMT1), also known as G9a-like protein (GLP), specifically dimethylates H3K9. Balemans and colleagues reported that knockdown of EHMT1 decreases H3K9me2 levels on the promoters of *Runx2* and *Col22a1* and thereby upregulates the transcription of these genes in brain and bone tissues. Although the authors did not provide direct evidence that EHMT1 regulates the osteogenic differentiation of MSCs, they found a severe type of craniofacial bone malformation, Kleefstra syndrome, in *Ehmt*^+/−^ mice [64]. In addition to HMTs, the HDM KDM4B plays a critical role in the osteogenic differentiation of MSCs by removing H3K9me3 [65]. Knockdown of KDM4B increases H3K9me3 on the promoters of several osteogenic genes and ultimately reduces osteogenic differentiation. Furthermore, a similar phenomenon has been observed in osteoporotic and aging mice, which may indicate a possible relationship between H3K9me3 and osteoporosis/senescence.

With regard to adipogenic differentiation of MSCs, G9a, an HMT that specifically methylates H3K9me1 and H3K9me2, has been proven to regulate PPARγ expression through enrichment of H3K9me2 on *Pparg* promoter regions and the treatment with BIX01294 (a G9a inhibitor) thereby promotes PPARγ expression and adipogenic differentiation [66]. In addition, the H3K9 demethylase PHF2 physically interacts with C/EBPα and C/EBPδ and binds to the *Pparg* and *Fabp4* promoters, ultimately promoting adipogenic differentiation by lowering the H3K9 methylation levels in these regions [67]. Inhibition of KDM4C, another H3K9 demethylase, with 2-hydroxyglutarate produced by mutating isocitrate dehydrogenase 1 (IDH1) blocks adipogenic differentiation [68]. Moreover, a recent report demonstrated that depletion of *JMJD1C* increases H3K9me2 levels on the *C/EBP* and *PPARG* promoters and suppresses adipogenesis by decreasing the expression of these master transcription factor genes [69]. Knockdown of SETDB1 also promotes adipogenesis through downregulation of H3K9me2 and upregulation of H3K4me2 on the *Cebpa* promoter [70]. Another study reported the existence of H3K4/H3K9me3 bivalent chromatin domains in the *Cebpa* promoter region and examined their regulatory effects on gene expression and adipogenesis. These H3K4/H3K9me3 bivalent chromatin domains are induced by DNA methylation and SETDB1 recruitment [71]. Similar to these domains, LSD1 also regulates H3K9me2 and H3K4me2 levels on the *Cebpa* promoter as well as on Wnt pathway gene promoters and promotes adipogenic differentiation by facilitating C/EBPα expression and inhibiting the Wnt signaling pathway [70, 72].

Research on AT-rich interactive domain 5b (Arid5b), a transcriptional coregulator of *Sox9*, has shown that this molecule promotes chondrogenic differentiation by recruiting the H3K9 demethylase PHF2 to the promoters of the *Sox9* target genes *Col2a1* and Aggrecan, thereby decreasing H3K9me2 levels in these regions [63]. Moreover, another HDM, KDM4B, removes H3K9me3 marks on the promoter region of *Sox9* and thus upregulates SOX9 expression [73]. Accordingly, depletion of KDM4B inhibits chondrogenic differentiation, while overexpression of KDM4B promotes chondrogenic differentiation.

#### H3K27 methylation

H3K27 methylation is another heterochromatic histone modification associated with transcriptional repression [74–76]. Wu and colleagues found that H3K27me3 levels are significantly reduced in the promoter regions of all transcriptionally upregulated genes during the process of osteogenesis [77]. Polycomb group (PcG) proteins, which were first identified 40 years ago, form PRC transcription-repressing complexes and are responsible for methylation of H3K27 [78]. Enhancer of zeste 2 (EZH2), a component of PRC2, increases H3K27me3 on the promoters of *Wnt* genes, including *Wnt1*, *Wnt6*, *Wnt10a*, and *Wnt10b* [79], suggesting a potential role of EZH2 in osteogenesis. Furthermore, knockdown of CDK-1, which phosphorylates EZH2 at T487, significantly increases EZH2 and H3K27me3 on the *Runx2* and *Tcf7* promoters [80]. Similar to knockdown of KDM4B, knockdown of KDM6B also attenuates osteogenesis by catalyzing H3K27me3 modification of osteogenic genes [65]. In addition, Hemming and colleagues demonstrated that KDM6A and EZH2 act as epigenetic switches regulating MSC differentiation. KDM6A and EZH2 regulate H3K27me3 status on the promoters of both osteogenic and adipogenic genes, including *Runx2*, *Oc*, *Pparg* and *Cebpa*, and knockdown of EZH2 or overexpression of KDM6A promotes osteogenesis [81]. A recent study further explored the roles of other novel EZH2-targeted genes in osteogenic differentiation and found that EZH2 binds to the TSS regions of *FHL1* and the osteogenesis regulators *ZBTB16* and *MX1* and establishes H3K27me3 modifications. Overexpression of EZH2 decreases the expression of these genes, which downregulates *RUNX2*, *OPN* and *OC* (Osteocalcin) [82].

EZH2 and KDM6A exhibit different regulatory functions in adipogenesis than in osteogenesis; a previous study revealed that overexpression of EZH2 or knockdown of KDM6A promotes adipogenic differentiation of MSCs [81]. However, this study did not elucidate the direct regulatory effects of EZH2/KDM6A or H3K27me3 modifications on adipogenesis-specific genes. A recent study showed that phosphorylation of H2B recruits EZH2 and initiates adipogenesis [83] through a mechanism involving increases in H3K27me3 levels at the promoters of *Wnt* genes and subsequent repression of *Wnt* gene expression.

In addition to affecting osteogenesis and adipogenesis, the H3K27me3 demethylases KDM6A and KDM6B have also been reported to facilitate the chondrogenic differentiation of MSCs [84–87]. A study on *Kdm6b*^−/−^ mice showed that KDM6B is recruited to the promoter of *Runx2*, a critical regulator of chondrocyte maturation and endochondral bone formation, and *Ihh* and activates the expression of these genes by decreasing the levels of repressive H3K27me3 marks [85]. Another study demonstrated that GSK-J4, an inhibitor of KDM6B and KDM6A, inhibits SOX9 and COL2A1 expression and thereby disrupts chondrogenic differentiation [84]. A further study showed that knockdown of KDM6A reduces H3K27me3 levels but increases H3K4me3 levels in the promoter regions of *Sox9* and therefore inhibits chondrogenic differentiation through repression of SOX9 expression. These results suggest that both KDM6A and KDM6B play essential roles in the entire process of chondrogenesis, from the initial stage of differentiation to the maturation of chondrocytes.

#### Other types of histone methylation

H3K36me3 is a histone mark that triggers transcription elongation [49]. SETD2, an HMT specifically catalyzing H3K36me3, is responsible for MSC osteogenic differentiation, and conditional depletion of *Setd2* in *Prx1*-Cre mice decreases bone formation. RNA-seq and ChIP-seq analyses have shown that loss of Setd2 results in downregulation of H3K36me3 levels on the *Lbp* gene and impairs the transcriptional initiation and elongation of this gene [88]. In addition, Lu and Colleagues demonstrated that the H3K36 mutations K36M and K36I impair MSC differentiation into chondrocytes and induce the formation of undifferentiated sarcoma [89]. Genome-wide profiling of H3K36me2/3 and H3K27me3 has shown that these mutations downregulate H3K36 methylation and lead to redistribution of the H3K27me3 landscape. The H3K36 mutation causes H3K27me3 to become enriched in intergenic regions and relatively scarce in genic regions, facilitating the expression of genes blocking mesenchymal differentiation. In addition to chondrogenic differentiation, knockout (KO) of the H3K36 methyltransferase Nsd2 has also been reported to impair adipogenic differentiation of MSCs by inhibiting PPARγ target gene expression [90].

The impact of H4K20 methylation on gene transcription is intriguing. H4K20 monomethylation is found in euchromatic regions and is associated with active transcription, while H4K20 dimethylation and trimethylation are more related to transcriptional repression and chromatin compaction [91–93]. SETD8, an HMT that specifically monomethylates H4K20, has been shown to participate in a positive feedback loop. SETD8 is upregulated by PPARγ during adipogenic differentiation, whereas SETD8 inversely modulates PPARγ expression through H4K20 monomethylation and thus promotes adipogenic differentiation of MSCs [94].

In addition to those on lysine residues, histone modifications on arginine residues also influence MSC differentiation. Coactivator-associated arginine methyltransferase 1 (CARM1), a protein arginine methyltransferase (PRMT) also known as PRMT4, targets histone H3 asymmetric methylation on arginine residues 17, 26 and 42 [95, 96]. Delivery of CARM1 into MSC nuclei using cell-penetrating peptides (CPPs) strongly increases the expression of pluripotent marker genes, including *Oct4*, *Sox2*, and *Nanog*, through upregulation of H3R17me2 levels on their promoter regions. In addition, CPP-CARM1 treatment enhances osteogenic, adipogenic and myogenic potential [95]. With regard to adipogenesis, another study has verified that CARM1 promotes adipogenic differentiation through coactivation with PPARγ [97]. CARM1 and PPARγ bind to the promoter region of *aP2*, an adipogenic gene, and induce *aP2* expression by upregulating H3R17 methylation [97]. PRMT5, another PRMT that mainly methylates H3R2, H3R8 and H2A/H4R3, has also been demonstrated to promote adipogenesis by upregulating adipogenic genes. Mechanistically, PRMT5 increases H3R8me2 levels on the adiponectin, resistin, and *aP2* promoters in C3H10T1/2 cells and then facilitates the expression of these genes via recruitment of BRG1, an ATPase subunit of SWI/SNF chromatin-remodeling enzymes [98].

#### Histone acetylation

HATs and HDACs are both essential for osteogenic differentiation. PCAF, an H3K9 acetyltransferase also known as KAT2B, is recruited to the promoters of *Bmp2*, *Bmp3*, *Bmpr1b* and *Runx2,* and knockdown of PCAF significantly impairs osteogenic differentiation of MSCs. In addition, osteoporotic mice show lower expression of PCAF in bone tissue than control mice [18]. GCN5, a paralog of PCAF also known as KAT2A, also contributes to osteogenesis. A recent study revealed that GCN5 binds to the promoters of *Wnt1*, *Wnt6*, *Wnt10a*, and *Wnt10b* and increases the expression of these genes through upregulation of H3K9ac levels. This activation of Wnt signaling results in enhanced osteogenesis [99]. Notably, some HATs function in a histone-independent way, regulating differentiation-related gene expression in MSCs and thereby fate commitment of MSCs through direct interaction with other non-histone proteins [100–103]. Several pan-HDAC inhibitors have also been reported to upregulate the osteogenic potential of MSCs [104–106], indicating the significant roles of HDACs in osteogenic differentiation. Furthermore, several studies have demonstrated the function of HDAC6 in the differentiation process [107–109]. The expression of HDAC6 is reduced during osteogenic differentiation, and HDAC6 negatively regulates the expression of *OC*, *Opn*, *Bsp2*, *Osx*, and *ALP* partly by binding to the *Runx2* C-terminus and adjusting *Runx2* activity. HDAC1 also affects osteogenic differentiation. Lee and colleagues reported that HDAC1 recruitment to the promoters of *Osx* and *OC* is reduced during osteogenesis of mouse BMSCs, leading to upregulation of H3K9 and H4 acetylation [110]. Furthermore, mechanical stimulation has been proven to affect the fate commitment of MSCs [111]. Wang and colleagues demonstrated that downregulation of HDAC1 during cyclic mechanical stretch (CMS)-induced osteogenic differentiation attenuates Notch signaling by inducing H3 acetylation on the promoter of *JAG1* [112]. In addition, the expression of *Col1a1* and *Runx2* has recently been shown to be decreased in chondrogenic cells from *Hdac3*–CKO_Prrx1_ mice in osteoinductive culture, indicating a supportive role of HDAC3 in osteogenic differentiation [113]. Another recent study found that upregulation of HDAC8 in fibrous dysplasia is associated with impaired osteogenesis, while HDAC8 inhibition promotes osteogenic differentiation of MSCs [114].

Depletion of CBP/p300 with ribozyme strongly represses the expression of PPARγ-targeted genes and thus attenuates adipogenic differentiation of preadipocytes [115]. Furthermore, studies on the mechanism of p300-mediated histone modification have revealed that p300 binds to *Pparg2* promoter and enhancer regions with enhanced H3/H4ac and H3K27ac, respectively [116, 117]. GCN5 and PCAF have also been reported to be involved in MSC adipogenic differentiation. These factors are recruited to *Pparg2* and *Prdm16* promoters and increase the H3K9ac levels on the sites. Depletion of both *Gcn5* and *PCAF* suppresses adipogenic differentiation of mice preadipocytes [118]. Yoo and colleagues [119] investigated the roles of HDACs in adipogenic differentiation and found that HDAC1, HDAC2 and HDAC5 are downregulated during adipogenic differentiation. Moreover, treatment with sodium butyrate (NaBu), a nonspecific HDAC inhibitor, promotes adipogenesis. The role of HDAC9 in adipogenic differentiation has been explored with *Hdac9*-KO transgenic mice [120]; such studies have revealed that HDAC9 is recruited to the *Cebpa* promoter along with USF1 and p300 and that KO of *Hdac9* promotes adipogenic differentiation of preadipocytes. However, a study on HDAC9 lacking the deacetylase domain showed that this effect of HDAC9 occurs through a deacetylase-independent mechanism. Although negative roles of HDACs in adipogenesis have been reported, Huang and colleagues proposed that HDAC6 facilitates adipogenic differentiation [108]. Their study revealed that inhibition of *Hdac6* with miR-22 represses adipogenic differentiation and relevant transcription factor expression but promotes osteogenic differentiation.

Tsuda and colleagues first reported the positive function of CBP/p300 in chondrogenesis [121], finding that CBP/p300 binds to *Col2a1* promoter regions in an interaction with Sox9 and promotes *Col2a1* expression as well as chondrogenesis. Another study revealed that p300 promotes *Col2a1* expression and chondrogenesis by increasing the histone acetylation levels of the *Col2a1* gene [122]. HDACs have also been reported to affect chondrogenic differentiation. For example, a study on the transcriptional regulation of cartilage oligomeric matrix protein (COMP), a marker gene for chondrogenesis, revealed a repressive role of HDAC1 in chondrogenic differentiation [123], demonstrating that leukemia/lymphoma-related factor (LRF) recruits HDAC1 to the negative regulatory element (NRE) of the COMP promoter and downregulates COMP expression. The inhibitory effect of the LRF-HDAC1 complex can be attenuated by application of Trichostatin A (TSA), indicating the critical role of HDAC1 in chondrogenesis. HDAC1 has also been reported to bind to the promoter of β-catenin and to suppress β-catenin expression, playing a negative role in chondrogenesis [124]. Similarly, HDAC4 functions negatively in chondrogenic differentiation [125]. Overexpression of HDAC4 attenuates chondrocyte hypertrophy and endochondral bone formation by inhibiting the activity of RUNX2, a regulator of chondrocyte hypertrophy. *Hdac4*-deficient mice show a phenotype with ectopic and early-onset chondrocyte hypertrophy, indicating upregulation of chondrogenic potential. In contrast, positive chondrogenic effects of the HDACs HDAC7 and HDAC3 have been identified by Bradley and colleagues [126, 127]. Depletion of *Hdac7* promotes chondrogenesis through upregulation of β-catenin. In addition, HDAC3 is recruited to the promoter of *Phlpp1*, a component regulating Akt signaling, and promotes chondrogenesis by repressing *Phlpp1* expression and upregulating Akt signaling. However, HDAC7 is highly expressed in proliferating chondrocytes, while HDAC3 is highly expressed in prehypertrophic chondrocytes, suggesting that different HDACs exert various functions at different stages of chondrogenesis.

### Roles of histone modifications in MSC transdifferentiation into other lineages

In addition to osteogenic, adipogenic and chondrogenic lineages, highly plastic and multipotent MSCs from different germ layers can differentiate into other cell lineages, such as neuron, epithelial cell, cardiomyocyte, hepatocyte and islet cell lineages, under certain induction culture conditions. Although the exact mechanism of transdifferentiation is largely unknown, epigenetic regulation has been reported to be critical in the process [3, 34]. In the next section, we discuss how histone modifications regulate the transdifferentiation of MSCs.

#### Neuronal differentiation

EZH2 is recruited to the promoter of *PIP5K1C*, a neuron-related gene regulating Ca^2+^ signaling, and downregulates *PIP5K1C* expression. Accordingly, knockdown of EZH2 promotes the neuronal differentiation of MSCs through activation of PIP5K1C-mediated Ca^2+^ signaling [128]. The G9a inhibitor BIX-01294 has been reported to promote neuronal differentiation. Mechanistically, BIX-01294 induces the expression of neuronal-specific genes, including Nestin, Musashi, *CD133* and *GFAP*, by downregulating *G9a* as well as H3K9me2 levels at repressor element-1 (RE-1) of these genes [129]. In addition to histone methylation, histone acetylation has also been shown to affect neuronal differentiation; the HDAC inhibitor valproic acid (VPA) facilitates MSC neuronal differentiation by upregulating neuronal-specific genes [10]. These results have been further validated with human placenta-derived MSCs and have been proven to be mediated by inhibition of HDAC2 [130]. Recently, HDAC inhibitors including MS-275, NaBu, TSA, and VPA were reported to promote neuronal differentiation of MSCs through activation of the Wnt signaling pathway [131].

#### Cardiomyogenic differentiation

Suberoylanilide hydroxamic acid (SAHA), an HDAC inhibitor, is much more powerful than 5-azacytidine, a DNA methylation inhibitor, in inducing the expression of the early cardiomyocyte-specific genes *GATA4* and *Nkx2.5*, indicating that histone acetylation levels are possibly more vital than DNA methylation levels during cardiomyogenic differentiation [132]. GCN5 plays a favorable role in cardiomyogenic differentiation [133, 134]. GCN5 induces H3 acetylation on the promoters of *GATA4* and *Nkx2.5* and enhances the expression of these genes under cardiomyogenic induction conditions [133]. In addition, knockdown of HDAC1 facilitates the expression of the cardiac-specific genes *GATA4*, *Nkx2.5*, *CTnT*, and *MHC*, thereby promoting cardiomyogenic differentiation [135]. These findings have also been validated by Wang and colleagues [9], who demonstrated that knockdown of HDAC1 or *Hdac2* enhances the expression of the cardiomyocyte-specific genes *Myh6* and *Tnni3* through upregulation of both H3 and H4 acetylation levels on the corresponding promoters.

#### Endothelial differentiation

In addition to cardiomyogenic differentiation, HDAC inhibitors can also promote hepatic differentiation of MSCs [136, 137]. Snykers and colleagues revealed that TSA supplementation greatly enhances hepatic differentiation induced by hepatogenic factors [136]. The resulting differentiated cells exhibit epithelial morphology, hepatic gene expression and hepatocyte functions. In addition, VPA promotes hepatic differentiation of MSCs by upregulating global H3/H4 acetylation levels [138]. In addition to hepatic lineage cells, MSCs can transdifferentiate into cells of another endoderm cell lineage: endothelial cells. Inhibition of G9a with BIX-01294 promotes endothelial differentiation of human adipose-derived MSCs by enhancing the expression of some endothelial markers and blood vessel formation factors [139].

## Roles of histone modifications in MSC senescence

The numbers of MSCs isolated from tissues are usually insufficient to meet clinical needs. Therefore, these primary MSCs are usually expanded in vitro. However, in vitro culture changes the morphology and reduces the differentiation potential and proliferation ability of late-passage MSCs, while in vivo senescence disrupts tissue homeostasis and eventually leads to aging-related diseases [140, 141]. Mechanistically, various cell biological processes contribute to the senescence of MSCs, including telomere- and telomerase-related processes, epigenetic changes, gene expression changes and immunological processes [142]. Among the alterations, alterations in histone modification patterns have been shown to be associated with senescence in studies on different species and cell lineages [143]. Correlations between histone modifications and DNA methylation in the context of MSC senescence are elucidated by a genome-wide study [144]. The results show that DNA-methylated genes display certain tendencies toward hyper- or hypomethylation during aging. In addition, hypermethylation is associated with the repressive histone marks H3K9me3 and H3K27me3, while hypomethylation is associated with H3K4me1.

During cellular senescence, the repressive histone modification H3K27me3 is downregulated, indicating its vital role in aging [145, 146]. Recently, Li and colleagues demonstrated that deletion of *Ezh2* results in premature senescence of MSCs in a study on *Ezh2*-CKO_Nestin_ transgenic mice [147]. Aged MSCs, marked with a Nestin-GFP^−^ signature, express much less EZH2 than control MSCs. Downregulation of *Ezh2* decreases H3K27me3 marks on the promoters of *p15*^*INK4b*^, *p16*^*INK4a*^, the cell cycle inhibitor genes *p21*^*CIP1*^ and *p27*^*KIP1*^ and facilitates the expression of these genes. Furthermore, KO of *Ezh2* increases the percentage of SA-βGal^+^ cells, directly indicating that cellular senescence is upregulated.

Another HMT, SUV39H1, which specifically targets H3K9me3, also functions in MSC senescence [148]. One study on a premature aging model demonstrated that KO of *WRN* in MSCs decreases H3K9me3 on *α*-*Sat* and *Sat2* loci and upregulates the transcription of these genes. Knockdown of SUV39H1 or HP1α, a cofactor of SUV39H1, results in downregulation of global H3K9me3 levels and MSC senescence, while overexpression of HP1α upregulates H3K9me3 levels and therefore represses MSC senescence. Similarly, MSCs with catalytically inactivated endogenous SUV39H1 exhibit the same phenotype of accelerated cellular senescence, indicating that histone modifications induced by SUV39H1 and HP1α play significant roles in MSC senescence.

With regard to histone acetylation, Li and colleagues found that the expression of RUNX2 and ALP increases in MSCs during aging, whereas that of the stemness markers OCT4 and SOX2 decreases. These alterations in gene expression result from dysregulation of H3K9ac and H3K14ac marks in the promoter regions of these genes, while methylation of CpG islands appears to remain unchanged [149]. As observed in previous studies [150–152], aging MSCs progressively lose their proliferation and differentiation potential, although osteogenic ability is attenuated more rapidly than adipogenic ability. A recent study revealed that the levels of the H3K9 acetyltransferase PCAF are significantly decreased in aged mice, which increases adipogenesis and decreases osteogenesis. Reducing PCAF in aged mice represses the expression of some osteogenic genes through downregulation of H3K9ac levels on these genes [18]. In contrast, another study on changes in adipogenic potential in aged MSCs demonstrated that recruitment of BMI1 and EZH2 to the promoters of adipocyte-specific genes results in high H3K27me3 levels, repressing the expression of these genes and therefore inhibiting adipogenic differentiation [153].

HDACs seem to exert various influences on MSC senescence. One study found that MSC senescence decreases the expression of HDAC1 and HDAC2. HDAC1 and HDAC2 upregulate the expression of HMT PcGs such as BMI1, EZH2 and SUZ12 through RB phosphorylation while downregulating the expression of KDM6B through direct promotion of H3 acetylation on the KDM6B promoter [154]. As a result, inhibition of HDAC1 and HDAC2 with specific siRNAs decreases PcG expression and increases KDM6B expression, reducing the levels of repressive H3K27me3 marks on the *p16*^*INK4A*^ promoter and initiating expression of this gene [154]. In contrast, Zhu and colleagues found that HDAC4, HDAC5 and HDAC6 are increased during MSC senescence. This change is associated with decreases in global H3 and H4 acetylation levels and OCT4 expression [155]. The HDAC inhibitors TSA and largazole enhance the expression of some pluripotent and proliferative genes through upregulation of H3K9ac and H3K14ac and therefore delay the senescence of MSCs [156]. However, NaBu, VPA and MS-275 enhance MSC senescence [154, 157]. The conflicting effects of these pan-HDAC inhibitors on MSC senescence may be due to different effects on different HDACs.

## Roles of histone modifications in other MSC biological processes

The therapeutic potential of MSCs is associated with their paracrine effects [158], and regulation of the HAT GCN5 could amplify angiogenesis during MSC therapy [159]. In osteoporosis mice, decreases in GCN5 are associated with impairment of the proangiogenic capacity of BMSCs. Mechanistically, GCN5 binds to the promoter of *Vegf* and increases H3K9ac levels, which facilitates VEGF expression and therefore enhances angiogenesis. Shen and colleagues found that the lncRNA H19 affects angiogenesis of human amniotic MSCs (HAMSCs) in an EZH2-dependent manner [160]. Knockdown of H19 in HAMSCs inhibits the formation of vessel‐like structures when HAMSCs and human umbilical vein endothelial cells (HUVECs) are injected subcutaneously into nude mice. Mechanistically, H19 interacts with EZH2, facilitating its binding to the promoter region of VASH1, an angiogenesis inhibitor gene, and therefore suppresses VASH1 expression by increasing H3K27me3 levels.

Efforts have been made to promote the therapeutic effects of MSCs through genetic modification and pretreatment (priming) before MSC administration [161–164]. For example, treatment with combinations of DNA-hypomethylating agents and HDAC inhibitors has recently been shown to enhance the anti-inflammatory effects of MSCs [165]. Such epigenetically modified MSCs express increased levels of IL-10 and IDO and inhibit T cell differentiation of peripheral blood mononuclear cells (PBMCs) under T0 and T17 conditions. These MSCs also suppress the expression of proinflammatory factors, including IL-17, IFN-γ and IL-2, when cultured with PBMCs. Furthermore, coculture of epigenetically modified MSCs with synovial fluid mononuclear cells (SFMCs) from rheumatoid arthritis patients decreases IL-17^+^/CD4^+^ T cell populations and downregulates IL-17 and IL-2 expression. Sphingosine-1 phosphate (S1P) is used as a priming factor in MSC therapy to enhance MSC function; however, S1P exhibits low efficacy and can induce adverse inflammatory reactions in vivo [166]. A recent study has reported that the HDAC inhibitor VPA can cooperate with S1P to increase CXCR4 expression in MSCs and to enhance MSC migration, self-renewal and anti-inflammatory capabilities [167].

Furthermore, HDAC1 silencing in human umbilical cord-derived MSCs (hUC-MSCs) enhances the neuroprotective effects of these cells against traumatic brain injury (TBI) in mice [168]. Knockdown of HDAC1 in hUC-MSCs enhances cell viability and attenuates oxidative stress, neuroinflammation and cell death after TBI through modulation of the PI3K/AKT pathway. However, how HDAC1 regulates PI3K/AKT on the chromatin level remains unclear.

## Conclusion

Through this discussion of various fruitful studies, we have provided a brief overview of the roles of histone modifications in MSC biology. Modulation of histone-modifying enzymes is likely a promising strategy for regulation of MSC differentiation and senescence.

However, the targets of histone-modifying enzymes seem to be relatively nonspecific; thus, manipulating these enzymes could influence more than one cell function. For instance, KDM4B and KDM6B regulate RUNX2 and SOX9 expression, which may facilitate both osteogenic and chondrogenic differentiation. Inhibition of G9a promotes adipogenic, endothelial, neuronal and cardiomyogenic differentiation. Similarly, EZH2 facilitates both adipogenic and neuronal differentiation and is also associated with cell senescence.

Therefore, precise, gene-specific control of certain histone-modifying enzymes remains a considerable challenge. However, there are a few possible directions for future research. First, a deeper and more systematic understanding of histone modification would help elucidate the gene-specific control of histone modification enzymes. In addition to more studies on individual histone modification marks or enzymes, comprehensive studies on the interactions of sequence-specific transcription factors with known histone modification systems are of great necessity and importance. Second, since some drugs or small molecules targeting histone-modifying enzymes have already been discovered and are even undergoing clinical trials [169–172], cocktails of several kinds of drugs should be investigated, as these might help enhance the specificity and efficiency of the existing compounds. Finally, coordination of the histone modification system with genome-editing techniques using CRISPR/Cas9 [173] and other vectors [174] is a promising strategy for precise regulation.

In summary, histone modifications undoubtedly play important roles in the regulation of biological processes in MSCs. Precise control of these modifications in MSCs will not only help us understand their functions but also enable us to direct cell fate for optimal tissue regeneration and damage repair.

## Data Availability

Not applicable.

## Abbreviations

ARDS: Acute respiratory distress syndrome
Arid5b: AT-rich interactive domain 5b
ASCOM: ASC-2 complex
BMSCs: Bone marrow stem cells
C/EBPα: CCAAT/enhancer-binding protein alpha
CARM1: Coactivator-associated arginine methyltransferase 1
CMS: Cyclic mechanical stretch
COMP: Cartilage oligomeric matrix protein
CPPs: Cell-penetrating peptides
EHMT1: Euchromatin histone methyltransferase 1
ESCs: Embryonic stem cells
EZH2: Enhancer of zeste 2
GLP: G9a-like protein
GvHD: Graft-versus-host disease
H3K27: Histone H3 lysine 27
H3K36: Histone H3 lysine 36
H3K4: Histone H3 lysine 4
H3K9: Histone H3 lysine 9
H3R17: Hisotne H3 arginine 17
HAMSCs: Human amniotic mesenchymal stem cells
HAMSCs: Human amniotic MSCs
HATs: Histone acetyltransferases
HDACs: Histone deacetylases
HDMs: Histone demethylases
HMTs: Histone methyltransferases
hUC-MSCs: Human umbilical cord-derived mesenchymal stem cells
HUVECs: Human umbilical vein endothelial cells
IDH1: Isocitrate dehydrogenase 1
iPSC: Induced pluripotent stem cells
ISCT: International Society for Cellular Therapy
KDM2A: Lysine-specific demethylase 2A
KDM4B: Lysine-specific demethylase 4B
KDM5A: Lysine-specific demethylase 5A
KDM5B: Lysine-specific demethylase 5B
KDM6A: Lysine-specific demethylase 5A
KDM6B: Lysine-specific demethylase 5B
KO: Knockout
LRF: Leukemia/lymphoma-related factor
MLLs: Mixed lineage leukemias
MSCs: Mesenchymal stem cells
NaBu: Sodium butyrate
NRE: Negative regulatory element
OA: Osteoarthritis
OC: Osteocalcin
OFCD: Oculofaciocardiodental
OSX: Osterix
PBMCs: Peripheral blood mononuclear cells
PcG: Polycomb group
PPARγ: Peroxisome proliferator-activated receptor gamma
PRMT: Protein arginine methyltransferase
RE-1: Repressor element-1
RUNX2: Runt-related transcription factor 2
S1P: Sphingosine-1 phosphate
SAHA: Suberoylanilide hydroxamic acid
SCAPs: Stem cells from apical papilla
SFMCs: Synovial fluid mononuclear cells
TBI: Traumatic brain injury
TSA: Trichostatin A
TSSs: Transcription start sites
VPA: Valproic acid
